# Integrating genetic and immune profiles for personalized immunotherapy in Alzheimer’s disease

**DOI:** 10.3389/fmed.2025.1603553

**Published:** 2025-06-02

**Authors:** Cong He, Yiwei Shen, Miao Zhang, Xiaoqing Zhou

**Affiliations:** ^1^Second Clinical Medical College, Heilongjiang University of Chinese Medicine, Harbin, China; ^2^Shenzhen Hospital of Beijing University of Chinese Medicine (Longgang), Shenzhen, China

**Keywords:** Alzheimer’s disease, personalized immunotherapy, genetic-immune integration, biomarker identification, precision treatment

## Abstract

Alzheimer’s disease (AD) is the most frequent cause of dementia worldwide, and it is estimated that the number of patients will increase to 131 million by 2050. Most of the current methods of dealing with AD are designed to alleviate the symptoms, and there is no effective way of stopping the progression of the disease. Personalized immunotherapy has the potential to be highly effective and cut down on side effects because it can be targeted accurately and intervened early. Considering the genetic factors, many studies are increasingly looking at taking the immune status into account. This article further discusses the genetic and immune characteristics of AD, the methods of integrating multiple histological data, the identification of biomarkers, the stratification of patients, the precise treatment plans, and the application and future trends of immunotherapy, giving new directions for the future treatment of AD. In this mini-review, the authors address the critical role that genetic background and immune status play in shaping therapeutic strategies for AD, noting that there is a unique immune response in carriers of the APOEε4 allele compared to non-carriers, and that this difference may affect the course of the disease as well as the efficacy of immunotherapy. The aim of this review is to give an overview of the current understanding of the influence of genetic and immune factors on each other in AD, focusing on the impact of the APOEε4 allele on the immune response and its implications for immunotherapy.

## 1 Introduction

Alzheimer’s disease (AD) is the most common form of dementia, affecting approximately 47 million people worldwide, with projections indicating an increase to 131 million cases by 2050 (1–5). The growing prevalence of AD places a significant burden on healthcare systems and social support services, while also imposing substantial economic pressures (6). Core pathologic features of AD include the deposition of Aβ plaques in the brain and the hyperphosphorylation of tau protein in neurofibrillary tangles (7). Aβ is produced through abnormal cleavage by β- and γ-secretases, forming Aβ40 and Aβ42 monomers, which subsequently aggregate into amyloid plaques (8). These plaques, at high concentrations, trigger microglial infiltration and activation. However, microglia activation also plays a role in exacerbating neuronal injury through excessive immune responses and inflammation (9–12).

Current pharmacological treatments of AD are represented essentially by acetylcholinesterase inhibitors and the glutamate antagonist memantine (13). They inhibit acetylcholinesterase to reduce the breakdown rate of acetylcholine and therefore enhance central cholinergic neurotransmission (14). They may even exert an additional beneficial effect, such as delaying cognitive decline or improving functional activities in everyday life in the first year of treatment. However, they cannot impede the progress of the disease, and the cognitive functions continue to deteriorate rapidly after drug withdrawal (15–17). Besides, great variability concerning their efficacy is exhibited among different patients, and the risk of side effects associated with prolonged administration limits its long-term clinical application (18–20).

So far, immunotherapy aimed at controlling immune function has achieved striking successes in treating tumors, including but not limited to immune checkpoint inhibitors and CAR-T cell therapy (21–24). In AD, immunotherapy aims to stimulate the immune system to target and clear Aβ deposits, offering promise as a disease-modifying therapy (25). Personalized immunotherapy, in particular, holds significant potential for targeted precision, early intervention, and minimizing side effects (26–29). Early detection, facilitated by biomarker-based diagnostics, allows for timely interventions that may prevent or delay neuronal damage (30). Accordingly, tailored treatment plans in accordance with patient profiles, by adjusting dosages and treatment schedules, can ensure optimal immune response and reduction in side effects. This will definitely improve patient compliance and tolerance (31). By elucidation of the pathophysiological mechanism of AD, personalized immunotherapy will be incorporated into the benefits and innovations of genomics, proteomics, and other interdisciplinary disciplines that enable more appropriate and effective disease management (32). This review specifically examines the role of genetic and immune characteristics in AD, focusing on how the APOEε4 allele affects the immune response and disease progression, and we discuss how this knowledge can lead to the development of personalized immunotherapies that take into account individual genetic and immune differences, which can improve efficacy and cut down on adverse effects.

## 2 Genetic background and immune system in AD

APOE4 encoded by APOE ε4 is very different from APOE3 in lipid metabolism, resulting in disturbances of cholesterol transport and distribution in the brain, thereby affecting the stability and function of neuronal membranes (33). APOE4 is easier to bind with Aβ, promoting the aggregation and deposition of Aβ to form amyloid plaques, which is one of the typical pathologic features of AD. The interaction of APOE4 with mitochondria triggers mitochondrial dysfunction, which is manifested as increased mitochondrial calcium levels and increased reactive oxygen species generation, further aggravating energy metabolism disorders and oxidative stress and impairing neuronal survival (34). From the perspective of the immune response, compared with other APOE isoforms, APOE4 is more susceptible to proteolytic hydrolysis under stress conditions, and the generated products promote neurofibrillary tangle formation, affect the function of immune cells including microglia, and decrease the efficiency of Aβ clearance, thereby contributing to AD. APOE4 is able to translocate into the nucleus, where it drives the expression of genes involved in senescence, Aβ production, inflammation, and apoptosis, further exacerbating the pathologic changes in AD (35–39).

**FIGURE 1:**
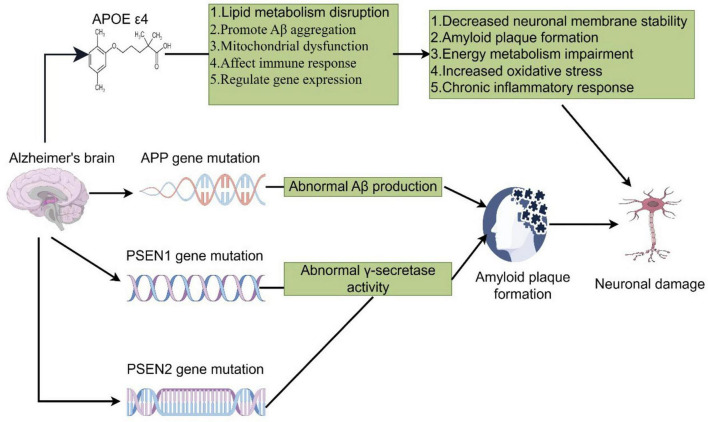
The APOEε4 allele disrupts lipid metabolism, promotes Aβ aggregation, impairs mitochondrial function, alters immune responses, and regulates gene expression. These changes further lead to the formation of amyloid plaques, oxidative stress, impairment in energy metabolism, chronic inflammation, and neuronal damage. Mutations within the APP, PSEN1, and PSEN2 genes promote abnormal Aβ production and γ-secretase activities, thus acting to further promote the amyloid plaque formation and neurodegeneration (by Figdraw).

Familial Alzheimer’s Disease (FAD) is primarily caused by mutations in the APP, PSEN1, and PSEN2 genes, which encode proteins critical to Aβ generation and clearance (40, 41). Mutations in the APP gene result in the abnormal accumulation of Aβ following the cleavage of amyloid precursor protein by β- and γ-secretases at the cell membrane, thus promoting the formation of amyloid plaques (42, 43). The PSEN1 and PSEN2 genes encode the key components of the γ-secretase complex, and mutations in these genes lead to abnormal γ-secretase activity, increasing Aβ generation and deposition. Notably, mutations in the PSEN1 gene are the most common pathogenic factor in FAD (Figure 1) (44–47). While the occurrence of PSEN2 gene mutations is seldom observed to be causing AD, it functions much like PSEN1; that is, Aβ tends to accrue abnormally.

Genes regulate the intensity of immune responses by influencing the development and differentiation of immune cells, as well as by modulating cellular signaling pathways (48–50). Studies suggest that the TREM2 gene, expressed in microglial cells, plays a crucial role in regulating their phagocytic function and inflammatory response. By contrast, PSEN1 mutations modulate γ-secretase activity and hence Aβ generation and immune cell activation (51–54). Some of them induce an inappropriate expression of immune markers and lead or maintain inflammatory responses. For example, APOE ε4 allele is associated with higher levels of inflammation markers such as IL-6 and TNF-α whereas mutations in TREM2 impact the inflammatory response in microglial cells (55–58).

AD involves a dual function of microglia and astrocytes in the pathologic process. These glial cells promote tissue repair and maintain neurological homeostasis through clearing of Aβ plaques and neurofibrillary tangles, secretion of anti-inflammatory cytokines such as IL-10 and TGF-β (59–61). On the contrary, over-activated microglia and astrocytes lead to the chronic inflammatory response that results from releasing a huge amount of pro-inflammatory cytokines such as TNF-α and IL-1β (62–64). These pro-inflammatory factors, while promoting further neuroinflammation, causing neuronal damage and death, promote Aβ production and Tau proteins over-phosphorylation via activating several signaling pathways such as NF-κB, thereby setting up a vicious circle (65, 66).

## 3 Integration of genetic and immune profiles

### 3.1 Integration methods of multi-omics data

**FIGURE 2:**
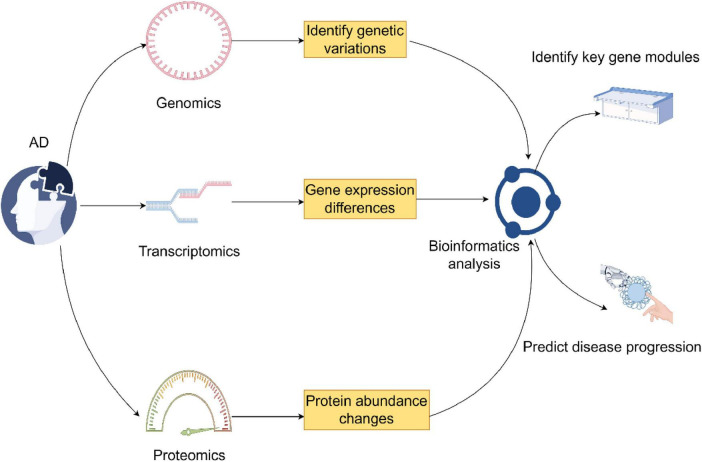
This figure combines genomics, transcriptomics, and proteomics in the investigation of Alzheimer’s disease. It identifies genetic variations through genomics, transcriptomics through differences in gene expression, and changes in protein abundance through proteomics. Bioinformatics analysis links them in the identification of the key gene modules and prediction of the disease outcome (by Figdraw).

In this respect, GWAS in genomics found genetic variants that are associated with AD and definitely confirmed that APOE ε4 is a major genetic risk of the disease. Besides regulating Aβ metabolism, these variants also enhance neuroinflammation by modulating the activity of immune cells (67–70). While transcriptomics offers information on dynamic gene expression changes, single-cell RNA sequencing has indeed enabled cell-type-specific investigation into AD-dependent shifts in gene expression at previously unparalleled resolution (71, 72). Indeed, microglia from AD brains have been found to express characteristic transcriptional phenotypes. It was noted that AD patients had a particular microglial transcriptional state in the brain that was strongly associated with inflammatory responses and lipid processing. Genomic and transcriptomic information is further complemented by proteomics, which examines protein expression and function, representing the end product of gene expression and their roles in diseased conditions (73, 74). Proteome-wide association studies integrate GWAS results with proteomic data to identify genes that influence AD risk by altering protein abundance. Bioinformatics tools and algorithms are thus very important for the integration of multi-omics data, enabling researchers to uncover more holistic insights into disease mechanisms (75). Weighted gene co-expression network analysis is one of the most popular methods used to identify gene modules associated with AD and their key hub genes. By constructing gene co-expression networks, researchers can pinpoint modules closely linked to disease progression and further explore their biological significance through functional annotation (76). Moreover, machine learning and artificial intelligence technologies have become crucial in the integration and analysis of multi-omics data (77). Approaches like deep learning can automatically identify patterns and trends from complex multi-omics datasets, enabling the discovery of novel biomarkers and the prediction of disease outcomes (Figure 2) (78).

### 3.2 Limitations of current omics technologies and barriers to implementation in clinics

Even with the significant development of multi-omics technologies, there are still some limitations and obstacles to their implementation in the clinical setting, one of which is the high cost and complexity of multi-omics technologies, which may affect their large-scale application in normal clinical practice (79, 80). Single-cell RNA sequencing, for example, contains a huge amount of information but requires specialized instrumentation and expertise, making it difficult to perform in small clinical laboratories. Furthermore, the sheer volume of data generated by these technologies requires sophisticated bioinformatics and computing resources, which are not readily available in all clinical settings (81). Another shortcoming is the lack of a standardized process for data collection, processing, and analysis, which can lead to different or even conflicting results from different studies or laboratories (82). Moreover, because of differences in data types, formats, and quality, fusion of multi-omics data from different sources may be challenging, requiring the creation of robust and flexible bioinformatics programs to ensure accurate and reliable data fusion (83). Translating the new insights from genomics into clinically feasible insights remains a major problem. Biological systems are complex and AD is highly variable, and it is difficult to identify clear and actionable biomarkers that can be used to make diagnoses, predict disease progression, and determine therapeutic response (84). The most common biomarkers are those that can be used for diagnostic purposes and that can be used in a clinical setting. Overcoming these limitations will require researchers, clinicians, and bioinformaticians to work together to develop more accessible, standardized, and clinically relevant genomic techniques and data analysis tools (85).

### 3.3 Identification and validation of biomarkers

High-throughput genomics technologies have made it possible to identify genes associated with AD and their expression pattern (86). Machine learning algorithms enhance the efficiency and accuracy of the analysis of gene data. By using machine learning algorithms like support vector machines and random forests, predictive models could be developed which could differentiate AD patients from healthy controls based on their sEV profiles (87–90). The immune system is a very important player in the pathology of AD. Inflammation markers, like sTREM2 and YKL-40, have been reported to be increased in AD patients and serve as biomarkers of neuroinflammation (91). Moreover, immune cell activation, especially microglia, is highly related to AD development, and proteins involved in immune signaling pathways can also be used as potential biomarkers. Immunomics analysis combined with machine learning methods could more effectively screen and validate immune-related biomarkers (92, 93). Further, it has been shown that plasma levels of p-tau181 provide high diagnostic accuracy for the early stages of AD (94, 95). Further sensitivity and specificity can be obtained by using an integrated diagnostic model that combines multiple biomarkers with clinical data. Such a model will help not only in the early identification of AD patients but also form a basis for developing personalized treatment plans, ultimately improving the prognosis and quality of life of patients (96, 97).

### 3.4 Patient stratification and precision therapy

APOE ε4 allele is the most important genetic risk factor for AD, and the patients carrying this allele have a higher incidence and faster development of the disease (98). The identification of such genetic alterations allows the division of patients according to different genetic subtypes and provides a rationale for personalized treatment approaches (99, 100). Inflammatory responses have been identified as one of the early features of AD and are closely related to disease progression. According to the immune cell types and levels of inflammatory factors, patients can be divided into different immune subtypes (101). High levels of pro-inflammatory factors, including IL-1β and TNF-α, indicate an active inflammatory process. Higher levels of anti-inflammatory factors, such as IL-10, reflect the patient’s immune regulation status (102). In patients with high-risk genetic mutations, lifestyle intervention, cognitive training, and other approaches may delay disease onset and progression. Personalized treatment plans should take both the patient’s genetic and immune profiles into account, often employing multi-target drug combination therapies (103). Given the long and complex course of AD, patient conditions change over time (104), necessitating dynamic adjustments to treatment plans based on ongoing assessments. Regular monitoring of genetic and immune biomarkers can provide critical insights into treatment efficacy and disease progression (105). Personalized treatment means the precise adjustment of medication doses and methods according to personal genetic background and medication metabolism capability, therefore (106).

### 3.5 Structural variability of Aβ and tau aggregates and its implications for personalized immunotherapy

Recent studies have shown that Aβ plaques and tau protein aggregates in the brains of AD patients have distinct individual and subtype-specific structural differences, a phenomenon known as “strain.” This conformational diversity alters the pathogenicity and spread of aggregates in the brain, and this structural change can make biomarker detection extremely difficult, as conventional diagnostic tests may not detect all forms of aggregates (107). From an immunotherapeutic perspective, antibodies targeting a specific conformation may not be effective in all patients, even to the extent that they do not work at all in those with different aggregation types (108). Therefore, future personalized immunotherapy strategies will have to take into account the biophysical properties of pathological aggregates, perhaps in combination with specific antibodies, aggregate sequencing technologies, and aggregate resolving imaging to achieve a higher degree of accuracy, which will also further subdivide patient stratification, thus driving the development of more effective and personalized therapies (109).

## 4 Current status and prospects of immunotherapy in AD

### 4.1 Types and mechanisms of immunotherapeutic approaches

Before elaborating on the types of immunotherapy for AD, it is important to clarify the fundamental differences between active and passive immunotherapy. Active immunotherapy relies on triggering the patient’s own immune system to recognize and fight against specific antigens, and this type of therapy often involves the use of vaccines or other medications that can trigger an immune response. Passive immunotherapy, on the other hand, refers to the injection of pre-prepared antibodies or other immune components directly into the patient, without the need for a response from the patient’s own immune system (110). These antibodies bind to and neutralize pathogenic proteins to reduce their harmful effects, and both approaches are aimed at improving the progression of AD, but they differ greatly in their principles of operation and possible side effects (111). The most important difference between the two approaches is that they have been shown to be effective in improving the progression of AD. Passive immunotherapy accelerates the process of pathologic protein removal through injecting certain monoclonal antibodies directly into a patient’s body. Aducanumab, Lecanemab, and Donanemab were permitted by the U.S. FDA for treating early AD (112). These antibodies delay cognitive decline by binding Aβ and promoting the removal of Aβ. Some adverse reactions, including brain edema and microhemorrhages, have been developed in clinical trials. Therefore, structural optimization and delivering strategy development to optimize safety and efficacy will probably be a more critical direction of the antibody-related therapy in future research (113).

Active immunotherapy, in contrast, elicits an immune response from the body by the administration of antigens or vaccines, which then clears the pathological proteins. Early research involving Aβ vaccines seemed promising, as vaccination in animal models led to a decrease in Aβ deposition and improved cognitive function. However, early clinical trials in humans were halted because some patients developed meningoencephalitis. With improved vaccine design and adjuvant selection, incidences of such side effects have been relatively rare in recent years (114). Several studies have demonstrated that vaccination against Aβ deposition, using antigens composed of non-Aβ peptides folded into a conformation similar to that of Aβ oligomers, effectively reduces Aβ deposition and improves cognitive function. Meanwhile, active immunotherapies do face some other major problems in clinical practices, including individual variation within immune responses, and the major call of inducing long-term immunity for many diseases (115, 116). Tau pathology is one of the key drivers of neurodegenerative, cognitive decline in AD, and an increasing number of studies are demonstrating the importance of tau pathology. Several monoclonal antibodies (semorinemab, gosuranemab, zagotenemab) have entered clinical trials that attempt to target extracellular tau proteins, but with mixed results (117). The results of this study have been reported in a number of studies. It is important to note that tau vaccines and antisense oligonucleotides (e.g., BIIB080) have the potential to reduce pathologic tau burden and slow cognitive decline in early trials, and other investigational treatments such as synaptic modulators, neuroprotectants, metabolic modulators, and anti-inflammatory drugs are changing the landscape of AD treatment (118). The current study of tau vaccines and antisense oligonucleotides has shown that they are effective in reducing pathologic tau burden.

**FIGURE 3:**
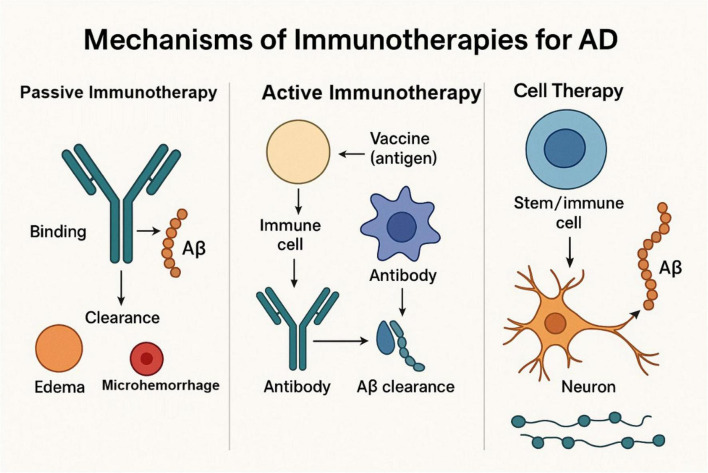
The mechanisms of three immunotherapies for AD. Passive Immunotherapy involves the direct administration of monoclonal antibodies to bind with Aβ for clearance, which may cause side effects such as edema and microhemorrhage. Active Immunotherapy stimulates the immune system through vaccination to produce antibodies that clear Aβ deposits, requiring optimized antigen design to reduce side effects. Cell Therapy uses stem or immune cell transplantation to repair damaged neural tissue or clear pathological proteins, although its clinical application is still in early stages. All three therapies target Aβ to slow the pathological progression of AD.

Cell therapies belong to a new direction in the field of AD treatment with great potential, involving the transplantation of certain cells with the aim of strengthening the immune response or repairing damaged neural tissues, the two main types of cell therapies undergoing research are listed below: CAR-T cell therapy involves engineering the patient’s T cells so that these T cells exhibit chimeric antigen receptors that specifically recognize and destroy cells bearing disease-associated antigens (Figure 3). In the setting of AD, CAR-T cells can be programmed to specifically target Aβ or tau proteins, and CAR-T therapies have had significant success in cancer treatment, although their use in AD is still in the early stages of research. Clinical studies have shown that CAR-T cells shrink Aβ plaques and optimize cognitive function in animal models (119) (Table 1). iPSCs can differentiate into a variety of cell types containing neurons and glial cells, which can be used as cellular replacement therapies, and iPSC-derived cells give the possibility of replacing damaged neurons and supporting neurologic repair. Numerous studies have shown that iPSC-derived neurons can be incorporated into the brain and improve cognitive function in animal models of AD to judge the safety and efficacy of iPSC-derived cell therapies in AD patients (120, 121).

**TABLE 1:** Therapeutic strategies for AD.

Therapy type	Mechanism description	Clinical application and challenges	References
Passive immunotherapy	Injection of monoclonal antibodiesto clear pathological proteins.	Approved for early AD treatment, optimization needed for safety.	(112)
Active immunotherapy (Vaccine)	Injection of antigens or vaccines to stimulate immune response.	Improved to reduce side effects, individual differences and immune memory maintenance are still challenges.	(114)
Cell therapy	Transplantation of immune cells or stem cells to repair neural tissue.	In early stages, further validation of safety and efficacy is required.	(122)

### 4.2 Review and analysis of clinical trials

Clinical Evaluation of Aducanumab: Primarily Two Phase III Studies-EMERGE and ENGAGE Both of these studies were designed as a randomized, double-blind, placebo-controlled, multicenter trials to evaluate the efficacy and safety of Aducanumab in early AD patients. The EMERGE and ENGAGE trials were designed to enroll thousands of patients with mild cognitive impairment and mild AD, respectively, for 78-week-long studies using variable dosing of Aducanumab for treatment, including high dosing at 10 mg/kg (123). In the EMERGE study, the high-dose Aducanumab group demonstrated a 30% reduction in the Clinical Dementia Rating-Sum of Boxes (CDR-SB) score at 78 weeks, a clinically significant symptom benefit. However, the ENGAGE trial failed to achieve the difference in the CDR-SB score statistically, comparing Aducanumab treatment and the placebo group. Potential causes may come from various aspects such as patients with baseline characteristics, different disease stages, and a progressive condition or even dosages during the treatment (124). The results of the EMERGE and ENGAGE trials have been the subject of much debate, particularly with regard to the U.S. Food and Drug Administration’s (FDA) granting of accelerated approval for Aducanumab, a pathway that permits approval of a drug on the basis of surrogate endpoints that may be indicative of clinical benefit, and which on the one hand is widely lauded for granting access to potential therapeutic treatments. On the other hand, it has been criticized for perhaps curtailing standards for drug efficacy and safety (125). Differences in the results of the EMERGE and ENGAGE trials have led to an enduring debate about the reliability of the accelerated approval process, as well as the need for more stringent post-marketing surveillance of Aducanumab’s long-term safety and efficacy (126). The results of the EMERGE and ENGAGE trials were not the same as those of the ENGAGE trial, which led to an enduring debate about the reliability of the accelerated approval process.

The Phase III clinical trial, Clarity AD, of lecanemab, in a double-blind randomized placebo-controlled fashion in approximately 2,000 patients with early-stage AD, was conducted. This single trial was designed to assess the effects of Lecanemab on cognitive function and Aβ deposition, with a fixed-dose, 18-month duration of treatment (127). The results of the Clarity AD trial revealed that Lecanemab was able to slow the rate of cognitive and functional decline by 27%, while significantly reducing Aβ levels in patients. Furthermore, lecanemab showed statistically significant benefit for three alternate cognition and function measures. Clinical benefits for Lecanemab in slowing the course of AD would therefore seem possible from these results (128).

The success of Lecanemab may be due to precision targeting and improved clinical trial design, where Lecanemab selectively neutralizes soluble and toxic Aβ aggregates, cuts down on non-specific binding, and reduces side effects, as well as a rigorous patient selection and stratification strategy that ensures credible trial results, factors that contributed to the success of Lecanemab in clinical trials (129). In contrast, the inconsistent results of the Aducanumab trial have generated much debate, and have demonstrated the importance of personalized treatment planning and rigorous trial design to prevent inconsistent efficacy due to patient heterogeneity, and that future clinical trials should incorporate genetic and immune profiling to better predict patient response and improve treatment outcomes (130).

### 4.3 Strategies and prospects of personalized immunotherapy

**FIGURE 4:**
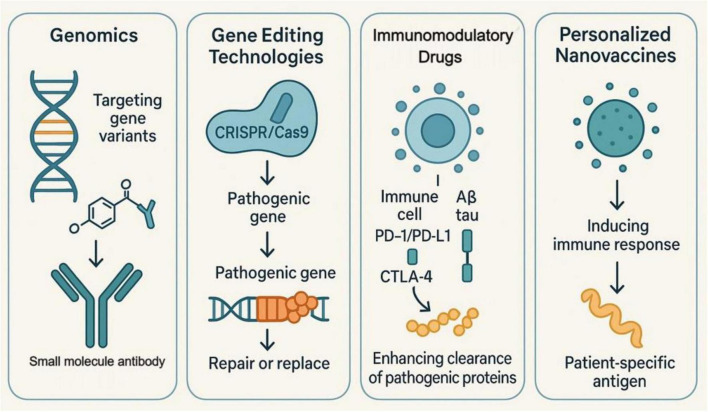
Four mechanisms of personalized immunotherapy in AD treatment. Genomics involves identifying gene variants associated with AD to design small molecule antibodies targeting Aβ and tau proteins. Gene editing technologies such as CRISPR/Cas9 enhance precision by repairing or replacing pathogenic genes, thereby reducing side effects. Immunomodulatory drugs, including PD-1/PD-L1 and CTLA-4 inhibitors, modulate the immune system’s response to pathological proteins, enhancing their clearance. Personalized nanovaccines induce an immune response using patient-specific antigens, improving the vaccine’s immunogenicity and specificity.

These advances in genomics have, in one way, helped to identify specific genetic variants linked with AD and have thus allowed the design of targeted approaches for treatment. In the case of genetic mutations in Aβ and tau proteins, specific small molecules or monoclonal antibodies can be developed. Moreover, new gene-editing technologies such as CRISPR/Cas9 offer new possibilities in the field of personalized gene therapy by allowing the direct repair or replacement of pathogenic genes. This approach not only increases the specificity of treatment but also reduces the destruction of normal cells, giving the patients more precise therapeutic options (131–134). Development of immune-modulating drugs is another important part of personalized immunotherapy. Very recently, immune checkpoint inhibitors including PD-1/PD-L1 and CTLA-4 inhibitors have shown tremendous achievements in several diseases. By modulating the immune response against Aβ and tau proteins, these drugs improve pathological protein clearance (135). Relevant studies have demonstrated that personalized nanovaccines, designed using tumor cell membrane vesicles from a patient, induce immune responses against patient-specific antigens. Such an approach enhances the immunogenicity and specificity of the vaccine, hence offering more effective options for the treatment of patients (136–138). Case studies related to integrated personalized strategies have pointed out that collaborative efforts and data sharing between disciplines hold immense potential to drive the development of personalized immunotherapies further. These have potential in the elucidation of AD with integrated genomic, proteomic data, and clinical information for personalized patient treatment options (139, 140). Establishment of a data-sharing platform: Clinical trial data, biomarkers, response to treatments-all this information can be shared on the data-sharing platform. This will help hasten the further development and validation of new therapies (141–143) (Figure 4). Further acceleration can be achieved by developing and validating new therapies through integration of patient genomic, proteomic data, and clinical information.

## 5 Conclusion and perspectives

In recent years, individualized immunotherapy has slowly become a key part of AD treatment. Individualized protocols based on each patient’s unique immune status and genetic profile can improve treatment efficiency and reduce side effects. Genetic situation and immune status play a very crucial role in the integration of AD salvage, and patients who carry the APOEε4 allele have a different immune response than non-carriers. Patient’s immune status also alters the efficacy of immune salvage. To better understand the mechanism of individualized strategies for AD immune salvage and to improve the salvage plan, there is an urgent need to explore in depth the effects of different genotypes and immune statuses on immune salvage response, and to combine the latest bioinformatics analysis techniques and AI algorithms, which are useful to facilitate the creation of accurate stratification and salvage prognostic models. Clinical trial design should be more individualized, and multicenter, large-scale clinical studies can be used to re-validate whether individualized immunotherapy is safe and effective. Regarding the future direction of research, we propose to carry out some specific actions, such as increasing the integration of multi-omics in clinical trials, forming biomarker clusters, adopting artificial intelligence and machine learning technologies, focusing on patient categorization and personalized trial design, implementing longitudinal observation and adopting adaptive trial design, and creating a platform for data sharing. It is also necessary to strengthen the search for basic research on genes and immune mechanisms, so as to give stronger theoretical support to the development of immunotherapy, and to pay attention to the study of the genetic backgrounds and immune characteristics of different races, so as to ensure the universality and usability of therapeutic strategies. In drug research and development, attention should be paid to the design and improvement of multi-target drugs to achieve all-round therapeutic results. In clinical practice, a long-term follow-up mechanism should be created to judge the long-term safety and efficacy of immunotherapy, thereby improving the quality of life of Alzheimer’s disease patients. For future research directions, we will pay special attention to the integration of multi-omics technology in clinical trials. Researchers should consider comprehensive multi-omics analysis as a standard procedure in clinical trials, including genomics, transcriptomics, proteomics and metabolomics, etc., so as to fully grasp the mechanism of disease and reflect the differences in the responses of individuals to treatments. Moreover, the integration of multi-omics data is useful for identifying new biomarkers and predicting disease outcomes more accurately, and the creation of multi-omics databases and data sharing platforms will enhance collaboration among researchers and accelerate the translation of research results into the clinic.
